# The microRNA expression profile of mouse Müller glia *in vivo* and *in vitro*

**DOI:** 10.1038/srep35423

**Published:** 2016-10-14

**Authors:** Stefanie G. Wohl, Thomas A. Reh

**Affiliations:** 1 Department of Biological Structure, University of Washington, School of Medicine, Seattle, WA, USA

## Abstract

The profile of miRNAs in mature glia is not well characterized, and most studies have been done in cultured glia. In order to identify the miRNAs in adult and young (postnatal day 11/12) Müller glia of the neural retina, we isolated the Müller glia from Rlbp-CreER: Stop^f/f^-tdTomato mice by means of fluorescent activated cell sorting and analyzed their miRNAs using NanoStrings Technologies^®^. In freshly isolated adult Müller glia, we identified 7 miRNAs with high expression levels in the glia, but very low levels in the retinal neurons. These include miR-204, miR-9, and miR-125–5p. We also found 15 miRNAs with high levels of expression in both neurons and glia, and many miRNAs that were enriched in neurons and expressed at lower levels in Müller glia, such as miR-124. We next compared miRNA expression of acutely isolated Müller glia with those that were maintained in dissociated culture for 8 and 14 days. We found that most miRNAs declined *in vitro*. Interestingly, some miRNAs that were not highly expressed in adult Müller glia increased in cultured cells. Our results thus show the miRNA profile of adult Müller glia and the effects of cell culture on their levels.

Müller glia (MG) are the primary intrinsic glia of the retina and share many features with astrocytes and radial glia in other regions of the nervous system, such as providing structural support and maintaining ionic homeostasis (for review see^1^,^2^). Another role for MG in non-mammalian vertebrates is to act as a source of new neurons after retinal injury. The MG in fish respond to retinal damage by re-entering the mitotic cell cycle and producing progenitor cells, similar to those present in embryonic development, and these cells go on to produce new retinal neurons. Several transcription factors, like Ascl1 and Pax6, are critical for the transition of MG to retinal progenitors (for review see^3^). More recently, miRNAs have been implicated in this process. In zebrafish, retinal damage leads to an increase in the RNA-binding protein, Lin28, and a subsequent reduction in the levels of the miRNA let-7. This reduction in let-7 expression is critical for regeneration in this species^4^. In addition, a Dicer knock down study in fish showed that miRNAs regulate proliferation of progenitors and are required for retinal regeneration after light damage^5^. Interestingly, MG from young mice can be reprogrammed to a neurogenic state with growth factors or transcription factors *in vitro* and *in vivo*^6^,^7^,^8^,^9^. In addition, we recently showed that miRNAs, i.e., miR-124, miR-9 and miR-9-5p, facilitate the production of neurons from mouse MG *in vitro*^7^. These studies from zebrafish and mouse show that miRNAs play some critical roles in regulating the state of the MG, and this motivated us to carry out an analysis of the miRNA profile of MG both *in vivo* and *in vitro*.

miRNA profiles have been generated for the neural retina^10^,^11^,^12^; however, these profiles represent the entire tissue and do not differentiate between different cell populations. Since MG comprise only 2–3%^13^ of the total retinal cells, we used fluorescence activated cell sorting (FACS) to purify adult MG, using an MG specific cre-lox labeling strategy by crossing Rlbp-CreER mice with stop^f/f^-tdTomato mice. The RNA was extracted from purified MG (tdTomato^+^) and retinal neurons (tdTomato^−^) and miRNA expression was analyzed by means of a recently developed molecular barcode technology called NanoStrings^®^^14^,^15^. Our analysis revealed a large number of miRNAs that were present in the MG samples, from both young mice (P11) and adults; some miRNAs were found to be specific to the MG (mGliomiRs, expression levels in neurons under 20%). Other miRNAs were expressed in both the MG and neurons (shared miRs) and others were specific to the neurons (neuronal miRs, expression levels in glia under 20%). Most of the MG-specific miRNAs increased during retinal maturation (from P11/12 to adult), but when compared with MG grown in dissociated cell cultures, about 80% of them were reduced substantially.

## Results

### miRNA profile of Müller glia and neurons in the adult mouse retina

**Figure 1:**
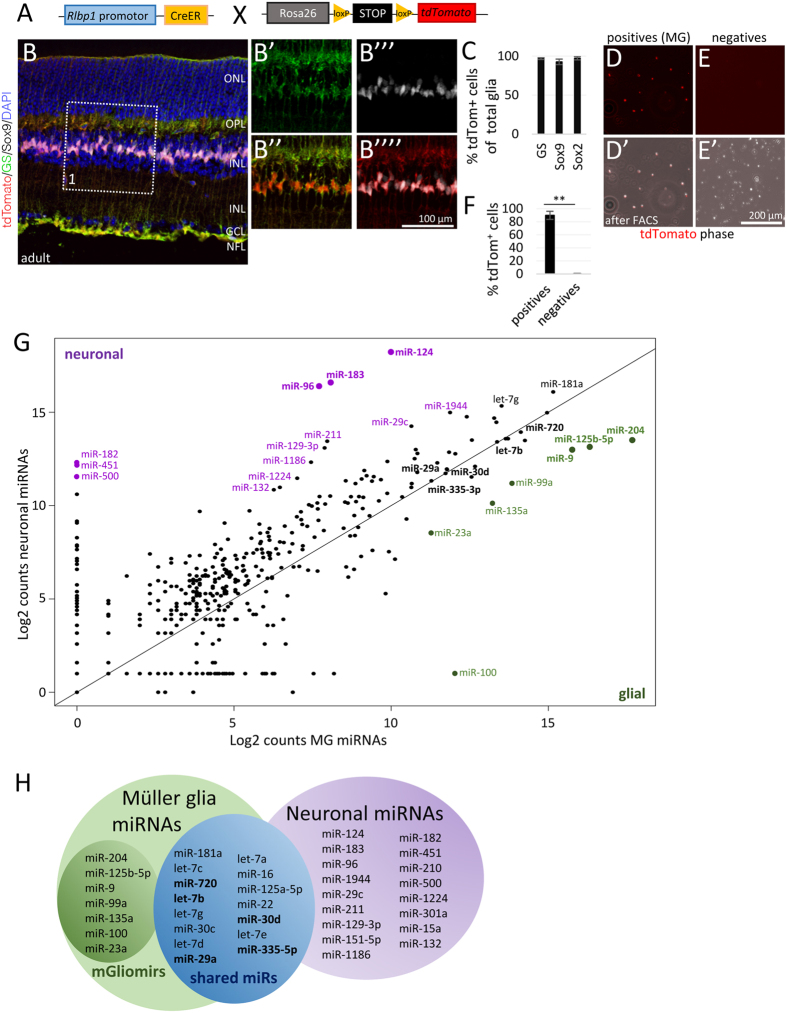
RlbpCreER: Stop^f/f^-tdTomato mice to isolate Müller glia (MG) and to identify glial and neuronal miRNAs. (**A**) Schematic of Rlbp-CreER: Stop^f/f^-tdTomato genotype. (**B**-B””_ Immunofluorescent stainings with antibodies against tdTomato (tdTom), glutamine synthetase (GS), and Sox2 as well as DAPI nuclear labeling. (**C**) Relative numbers of tdTomato^+^ MG co-expressing either GS, Sox9, or Sox2 of the total number of GS, Sox9 or Sox2^+^ glia in the INL, n = 6 mice. (**D,E**) Live images of plated tdTomato^+^ cells (MG, (**D**) D’) and tdTomato^–^ cells (neurons, (**E**) E’) after FAC-sort. (**F**) Quantification of the number of tdTomato^+^ cells found in the tdTomato^+^ (positives, MG) and tdTomato^–^ (negatives, neurons) fractions as percentage of the total number of cells, n = 13 mice, 3 different sorts. (**G**) Scatter plot of Log2 counts of the miRNAs from neurons and MG of adult retinas (n = 20) after NanoString analysis. All miRNAs expressed above the black line are miRNAs more highly expressed in neurons than in MG with the top neuronal miRNAs highlighted in purple. All miRNAs below the black line are miRNAs more highly expressed in glia than in neurons with the top 7 glial miRNAs (mGliomiRs) highlighted in green. miRNAs found on the black line have similar expression levels in neurons and MG. (**H**) Summary of the highly expressed miRNAs in either MG (green), neurons (purple) or both (blue). Significant differences are indicated, **p < 0.01, Student’s t-test for independent samples and Levene’s test for equality of variances. ONL: outer nuclear layer, OPL: outer plexiform layer, INL: inner nuclear layer, IPL: inner plexiform layer, GCL: ganglion cell layers, NFL: nerve fiber layer.

To determine which miRNAs are expressed in adult MG, we purified the MG from Rlbp1-CreER:Stop^f/f^-tdTomato mice (Fig. 1A) by FACS. We induced the reporter in the MG by tamoxifen injections and found that this resulted in tdTomato expression in the vast majority of MG, as assessed by Sox9 and Glutamine Synthetase (GS) co-labeling (Fig. 1B,C). Prior to FACS, the retinas were dissected free of pigmented epithelium and ciliary epithelium, were checked for successful recombination, then pooled and dissociated. The fraction of the tdTomato^+^ cells varied between 1.5–2.1% of all events (Supplement Fig. 1A-A”’, Supplement Table 1), consistent with previous estimates of MG numbers in the mouse retina^13^,^16^. Post-sorts of the tdTomato^+^ MG and the tdTomato^−^ fraction showed excellent purification (96% and 0.0% tdTomato^+^ cells of total cells, respectively, Supplement Table 1). To further assess potential contamination of the MG fraction with retinal neurons, a sample of the sorted cells was plated on coverslips and evaluated with fluorescent and phase microscopy: approximately 90% of all cells were tdTomato^+^, while no tdTomato^+^ cells were found in the negative fraction (Fig. 1D–F). Rlbp1 is also expressed in pigmented epithelial cells and cells of the ciliary epithelium^17^, and even though these were dissected from the retina, to ensure these were not included in our analysis, we plated the FAC-sorted adult cells onto coverslips and labeled the cells with antibodies against Sox9, Id1, and Sox2 to confirm the cell identity. We found that all tdTomato^+^ cells found in the P3-gated MG^+^ fraction were positive for all three markers while no red cells were found in the P2-gated tdTomato^−^ fraction (Supplement Fig. 1B–E). Since Sox2 is not expressed in the pigmented epithelium or the ciliary epithelial cells (Supplement Fig. 1F–H)^18^, these results confirm that over 95% of the cells used for subsequent analysis were MG. Moreover, to rule out the possibility that some rods might be attached to MG during the sort, we used the Nrl-GFP mouse^19^ in which all rod photoreceptors are GFP^+^ and crossed it with the Rlbp-CreER: Stop^f/f^-tdTomato mouse (Supplement Fig. 1I). We sorted the retinas of these mice with the same gating and plated the cells. There were no GFP^+^ rods in the wells with the tdTomato^+^ cells (Supplement Fig. 1J,K). These results demonstrate that FACS of MG produces a highly purified population.

To quantify the miRNAs expressed in MG, the total RNA was extracted from both the positive and negative fractions of the FACS purification, pooled from 40 retinas, and 600 miRNAs were quantified by solution hybridization without amplification using the NanoString nCounter^®^ assay^14^. An advantage of this approach is that the NanoString assay requires relatively small amounts of RNA (200 ng), which allows the analysis of small cell populations, and does not require amplification, which might introduce bias. The expression profiles of MG and neurons are shown in Fig. 1G as a scatter plot on a log2 scale: miRNAs expressed more highly in neurons than glia are above the line, while all the miRNAs expressed more highly in MG are below the line. Of 600 miRNAs analyzed, 23 showed expression higher than 2,000 counts in MG (Supplement Table 2). Of these 23, seven had more than 5 times greater levels of expression in MG than neurons, and we designate these as MG microRNAs: mGliomiRs (Fig. 1G green, H, Supplement Table 2, bold). The other 16 miRNAs of the highly expressed miRNAs in MG were also expressed in neurons to some extent (designated as “shared”). In the tdTomato^−^ neuronal population, we found 46 out of 600 miRNAs that were highly expressed (>2,000 counts, Supplement Table 3). Of these 46, 17 miRNAs had more than 5 fold higher expression levels in neurons than in MG and were designated as neuronal miRNAs (Fig. 1G purple, H, Supplement Table 3). The top three neuronal miRNAs were miR-124, miR-183, and miR-96, consistent with earlier reports demonstrating neuronal (for review see refs 20 and 21) and photoreceptor specific expression^12^,^22^,^23^,^24^,^25^.

**Figure 2:**
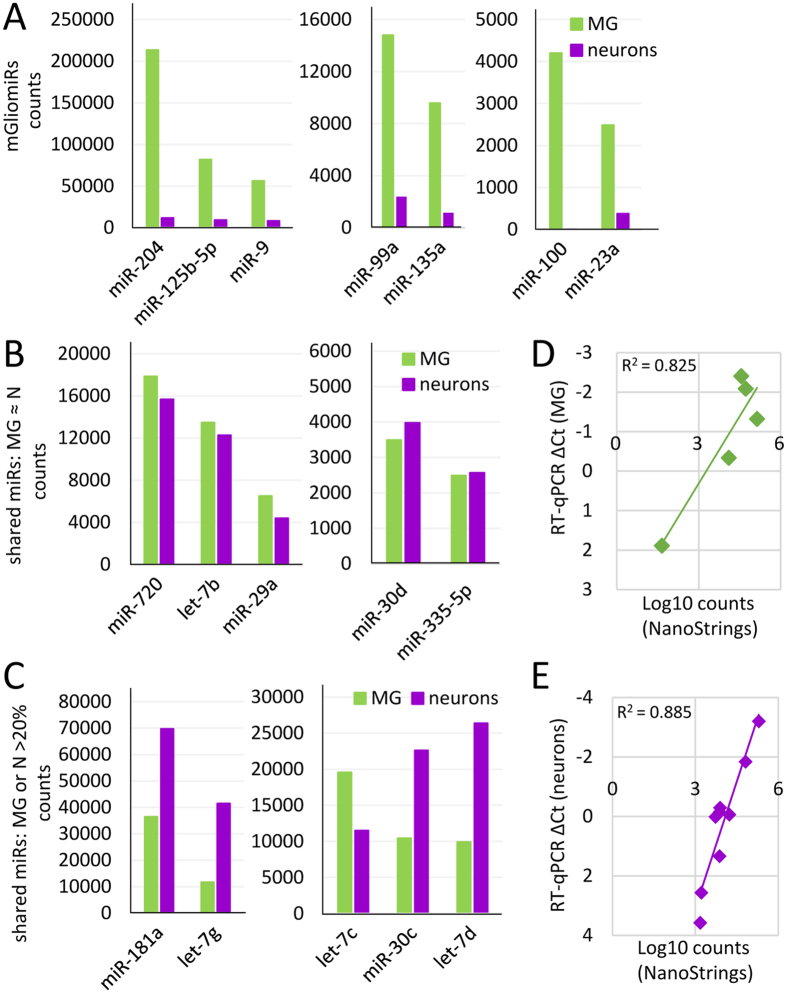
Highly expressed miRNAs of mature MG. (**A**) Top 10 Müller glia miRNAs (mGliomiRs) listed in order of their highest expression levels; notice different scale, cutoff 500 molecules; expression levels in neurons, displayed as purple bars, is less than 20%). (**B**) Graph of 8 out of 31 shared miRNAs with expression very similar expression levels in MG and neurons (MG ≈ neurons). (**C**) Graph of 5 shared miRs, found within the top 10 highly expressed miRNAs in MG, with expression levels in neurons or glia >20% and therefore excluded from mGliomiRs and neuronal miRs categories. (**D**) RT-qPCR validation of the 5 miRNAs miR-204, miR-125b-5p, miR-9, miR-181b and let-7c for the MG-enriched FAC-sorted fraction; comparison of ΔC_t_ means from FAC-sorted adult MG (normalized against beta-Actin and 5S) versus Log10 counts from NanoString^®^. Linear regression coefficient R^2^ = 0.825. (**E**) RT-qPCR validation of the 9 miRNAs miR-124, miR-183, let-7d, let-7b, miR-204, let-7a, miR-9, miR-15a and miR-30a for the neuronal FAC-sorted fraction; comparison of ΔC_t_ means from FAC-sorted adult MG (normalized against 5S) versus Log10 counts from NanoString^®^. Linear regression coefficient R^2^ = 0.885.

Figure 2 shows the expression levels of the different miRNA categories in the MG. The degree of enrichment of the mGliomiRs in our samples is displayed in Fig. 2A. miR-204, miR-125b-5p, and miR-9 are the top 3 mGliomiRs, and showed 18, 9 and 7 fold higher levels of expression in MG than in neurons. Similarly, the next most highly expressed mGliomiRs have at least 6 fold higher expression in MG than neurons, and miR-100 has less than 1% expression in neurons as compared to MG (Fig. 2A, Supplement Table 2). Another group of miRNAs that were highly expressed in MG were those shared between neurons and the MG. Five of these 15 shared miRNAs had comparable levels of expression in both the MG and neuronal fractions. These include miR-720, let-7b, miR-29a, miR-30d, and miR-335-5p (Fig. 2B, Supplement Table 2). Many of the other shared miRNAs had higher levels of expression in neurons than in MG; these include miR-181a, let-7g, miR-30c and let-7d; let-7c is the only miRNA in the shared group with higher expression levels in MG than in neurons, but does not fall in the mGliomiR category (expression levels in neurons >20%, Fig. 2C, Supplement Table 2). We validated the NanoString counts for several miRNAs for the MG-enriched and the neuronal FACS-fraction using RT-qPCR and there was a high degree of correlation (R^2^ = 0.825, Fig. 2D and R^2^ = 0.885, Fig. 2E).

### miRNA profile of Müller glia and neurons in the young mouse retina

**Figure 3:**
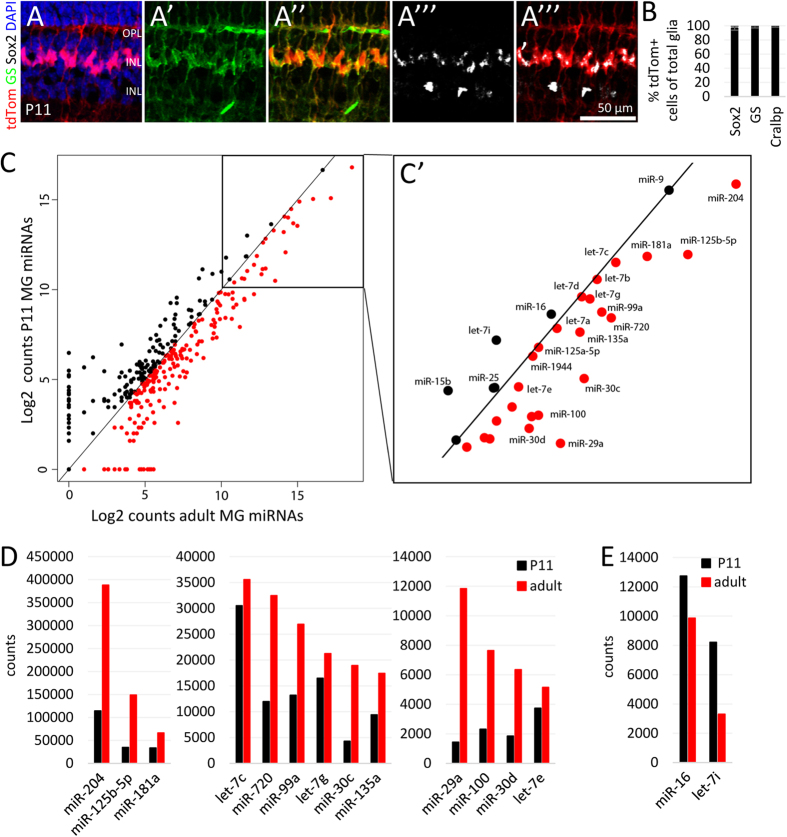
Most MG miRNAs increase with glial maturation. (**A-**A””) Immunofluorescent labeling with antibodies against tdTomato, glutamine synthetase (GS), Sox2, and DAPI nuclear staining. (**B**) Percentage of tdTomato^+^ cells coexpressing glial markers Sox2, GS, or Cralbp of total number of Sox2^+^, GS^+^ or Cralbp^+^ MG respectively, n = 4. **(C**) Scatter plot of Log2 counts of the miRNAs expressed in P11 MG (of 16 mice) and adult MG (of 20 mice). All miRNAs expressed above the black line are miRNAs expressed higher in P11 MG than in adult MG (black dots). All miRNAs below the black line are miRNAs higher expressed in adult MG than in P11 MG (red dots). C’ shows the boxed area of C with the 30 most highly expressed miRNAs. (**D**) Bar graph of 13 miRNAs (out of the 30 highly expressed miRNAs in C’), which expression levels further increased from P11 to adult (cutoff 5000 counts, increase >10%, notice different scales). (**E**) Bar graph of two miRNAs (out of the 30 highly expressed miRNAs in C’), in which expression levels declined from P11 to adult (cutoff 5000 counts, decrease >10%). OPL: outer plexiform layer, INL: inner nuclear layer, IPL: inner plexiform layer.

To evaluate if the miRNA profile differs between MG from young mice and adults, we sorted the MG from the P11 mice as described above, (Supplement Fig. 2A-A”’, Supplement Table 1). At P11, tdTomato^+^ cells have the morphology of MG, and express Sox2 and GS, like MG from adult mice (Fig. 3A,B). FACS purification of MG from the P11 mice resulted in approximately the same proportion of cells as in the adult. In order to check the purity of the tdTomato^+^ fraction, the cells were post-sorted; there was a high degree of purification (Supplement Table 1). To further verify the purification, a sample of the positive cells was maintained in cell culture for 5 DIV, and fixed and labeled for MG markers; we found that all tdTomato^+^ cells coexpressed glial markers, such as Id1 and Sox2. Moreover, cells expressing MG markers were not present in the tdTomato negative fraction, demonstrating that the neuronal fraction did not contain MG (Supplement Fig. 2B,J).

We subjected the RNA from MG purified from P11 mice to NanoString analysis as described for the adult MG. Nearly all of the miRNAs that were highly expressed in the MG of adult mice were also found in high levels in MG of P11 mice (R^2^ = 0.75). However, most miRNAs showed substantial increases in their level of expression between the young and mature MG. Figure 3C shows a scatter plot comparing the miRNA levels of the two ages. All the miRNAs that are more highly expressed in the adult MG are highlighted in red in the scatter plot, and the ones that showed increases of more than 10% (cutoff 5,000 counts) are plotted as bar graphs in Fig. 3D. Three of the top four miRNAs expressed in adult MG, miR-204, miR-125-5p and miR-181a, showed large increases in their expression between the P11 and adult, while miR-9, did not increase. In addition, many let-7 family members also increased between P11 and adult (Fig. 3D; Supplement Table 2). However, there were also miRNAs that declined as the MG mature, such as miR-16 and let-7i (cutoff 5,000 counts, decline >10%, Fig. 3E). These miRNAs might be relevant to earlier stages in MG development, since analysis of total retinal miRNA expression shows that they are more highly expressed in developing retina^10^.

### Changes of the miRNA profile of Müller glia *in vitro*

**Figure 4:**
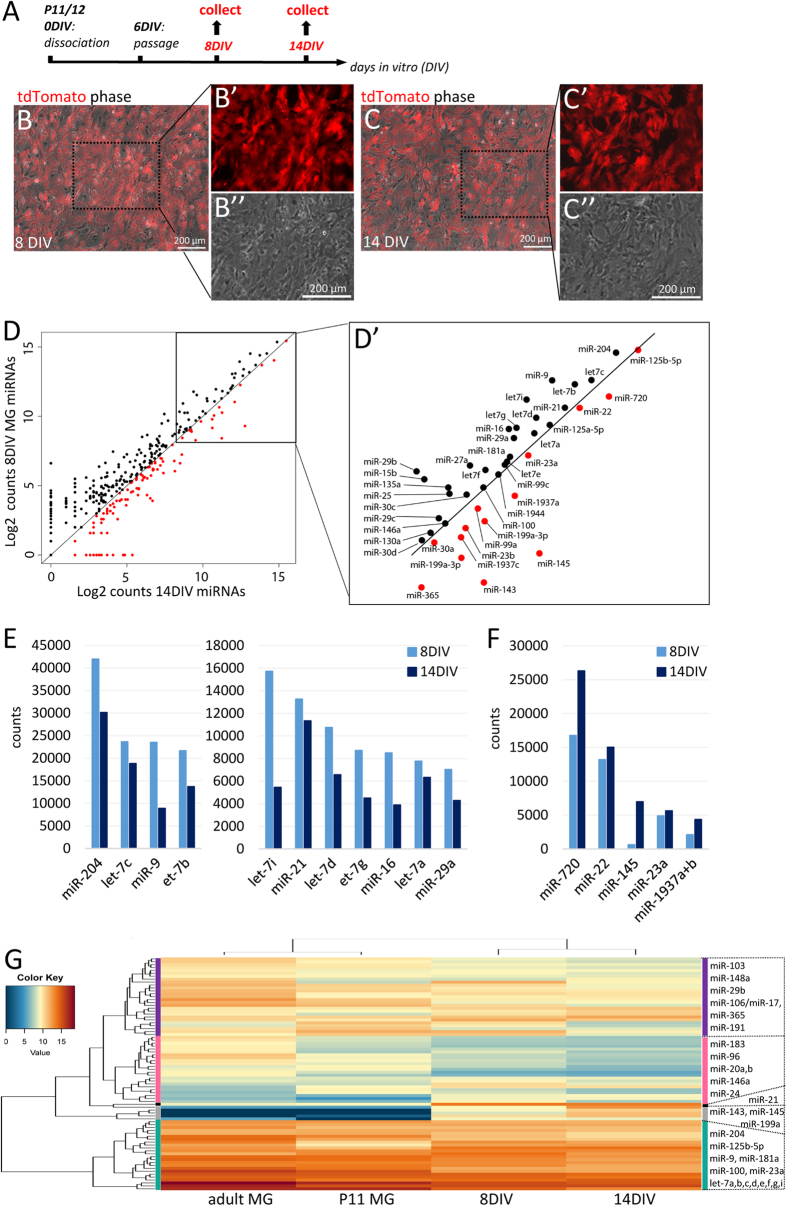
Most MG miRNAs decline *in vitro* over time. (**A**) Cell culture scheme. (**B,C**) Live images of tdTomato^+^ P11 MG 8 DIV (**B**-B”) and 14 DIV (**C**-C”). Boxes in (**B,C**) are shown in higher magnification in B’, B”, and C’, C” respectively. (**D**) Scatter plot of Log2 counts of the miRNAs expressed in MG 8 and 14 DIV. All miRNAs expressed above the black line are miRNAs expressed higher in 8 DIV MG than 14 DIV MG (declined over time, black dots). All miRNAs below the black line are miRNAs higher expressed in 14 DIV MG than in 8 DIV MG (increased over time, red dots). D’ shows the boxed area of C with the 42 most highly expressed miRNAs. (**E**) Bar graph of 11 miRNAs (out of the 43 highly expressed miRNAs in D’), which expression levels decreased from 8 DIV to 14 DIV (cutoff 5000 counts, decrease >10%, notice different scales). (**F**) Bar graph of five miRNAs (out of the 43 highly expressed miRNAs in D’), in which expression levels increased from 8 DIV to 14 DIV (cutoff 5000 counts, increase >10%). (**G**) Heat map showing hierarchical clustering of miRNAs and samples (see text for details).

A great deal of what is known about glia comes from *in vitro* studies^6^,^7^,^26^,^27^,^28^, and so we were interested in defining the miRNA profile of MG grown in dissociated cultures and comparing this with that of acutely isolated MG. In order to investigate miRNA expression *in vitro*, we prepared dissociated cell cultures of MG as previously described^6^,^29^. With this protocol, confluent monolayers of MG can be produced from P11/12 mouse retina and after a single passage at 6 DIV, few neurons remain (Fig. 4A). After passage, MG do not proliferate and over time cell density decreases (Fig. 4B,C). We harvested RNA from these cultures at either 8 DIV or 14 DIV, and processed the RNA for NanoString quantitation of miRNAs, similar to the acutely isolated samples.

Overall, the levels of miRNAs were similar in the two samples of MG that were maintained in culture (R^2^ = 0.9). Figure 4D compares the levels of miRNAs from the 8 DIV MG with those from the 14 DIV sample. The miRNAs more highly expressed at 8 DIV are shown in black, while those more highly expressed at 14 DIV are in red. The plot shows that most of the highly expressed miRNAs decline over the culture period, with lower levels of expression in the 14 DIV MG. The highly expressed miRNAs, that show the greatest changes are plotted in Fig. 4E,F (>5,000 counts, increase or decrease >10%). The miRNAs that decline the most over the culture period include miR-204, miR-9 and most members of the let-7 family. However, some miRNAs, like miR-720, miR-22, and miR-145, were substantially more highly expressed in the 14 DIV MG.

To compare the levels of miRNAs from MG maintained *in vitro* with those of the freshly isolated MG, we used a hierarchical clustering analysis (hclust; R). The results are shown as a heat map in Fig. 4G. Not surprisingly, the two samples from the MG maintained *in vitro* form a branch and the freshly isolated MG from the two ages comprise a second cluster. However, overall, the pattern of miRNA expression in the cultured cells and the freshly isolated cells is similar. The miRNAs are clustered into five groups: (1: turquoise) The most highly expressed miRNAs in all samples, which are primarily made up of the mGliomiRs and the shared miRNAs; (2, gray) a cluster of miRNAs not expressed *in vivo*, but with very high expression levels in the cultured MG; (3: black) a “cluster” with miR-21 alone, which is moderately expressed *in vivo* but increased substantially *in vitro*; (4: pink) a cluster with miRNAs expressed at low levels in the FAC-sorted MG and which decline further *in vitro* (including miR-146a, miR-20a+b) and lastly (5: purple) a cluster of miRNAs that are moderately expressed in freshly isolated samples and also decline *in vitro* (including miR-148a, miR-106/miR-17, and miR-191).

**Figure 5:**
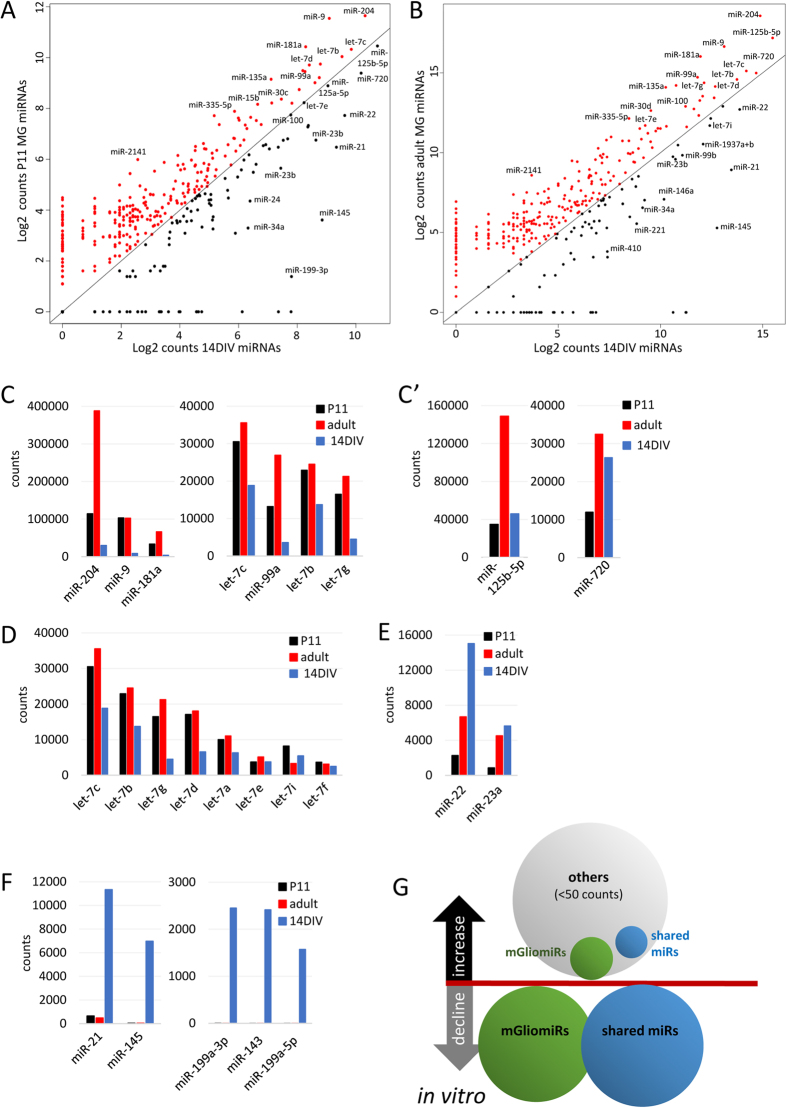
Comparison of the miRNA profile of MG *in vivo* and MG *in vitro*. (**A**) Scatter plot of Log2 counts of the miRNAs expressed in P11 MG and 14 DIV MG. The red dots represent miRNAs expressed higher in P11 MG *in vivo* than in 14 DIV MG, the black dots miRNAs more highly expressed in 14 DIV MG than in P11 MG *in vivo*. (**B**) Scatter plot of Log2 counts of the miRNAs expressed in adult MG and 14 DIV MG. The red dots represent miRNAs expressed more highly in adult MG *in vivo* than in 14 DIV MG, black dots miRNAs more highly expressed in 14 DIV MG than in adult MG *in vivo*. **C**,C’: Bar graphs of the top 9 miRNAs in which expression levels decreased after 14 DIV as compared to P11 or adult MG *in vivo* (**C**) or decreased as compared to adult MG but increased as compared to P11 MG (C’, bar graphs cutoff 20,000 counts, decrease >10%). (**D**) Bar graph of eight let-7 family members in P11 and adult MG *in vivo*, and MG maintained for 14 DIV. (**E**) Bar graph of two miRNAs in which expression levels increased after 14 DIV (bar graph cutoff 5,000 counts, increase >10%). (**F**) Bar graphs of 5 miRNAs in which expression levels increased substantially *in vitro*. (**G**) Summary of the changes in the miRNA profile of MG *in vitro* (data from 8 DIV and 14 DIV) as compared to MG *in vivo* (P11 and adult MG). Shown are the defined categories: mGliomiRs (high expression in MG with >2,000 counts, <20% expression in neurons) and shared miRs (highly expressed in MG with >2,000 counts but >20% expression in neurons or glia). miRNAs assigned as “others” are miRNAs not expressed in adult MG *in vivo* (<50 counts). The bubble size represents the percentage of the number of miRNAs that decline and/or decrease of the total number of miRNAs of the particular category, i.e., 72% mGliomiRs and 79% of the shared miRs declined *in vitro* as compared to the expression levels from freshly isolated adult MG; 28% mGliomiRs, 21% shared miRs, and 100% of other miRNAs increased *in vitro*. The range of increase or decline is given in the brackets.

The heat map also showed that the overall level of miRNA expression is lower in the cultured samples, when compared with the freshly isolated MG. This is shown more clearly in the scatter plots of P11 MG and MG cultured for 14 days (Fig. 5A). There were over twice as many miRNAs more highly expressed in the P11 sample (209) than in the 14 DIV sample (99). A similar relationship is seen when the adult MG miRNAs are compared with MG after 14 DIV (P11 + 14 DIV that corresponds to adult MG in age, Fig. 5B). The miRNAs more highly expressed in P11 or adult MG are highlighted in red. Interestingly, the decline in most miRNAs *in vitro* started already within the first week *in vitro* (Supplemental Fig. 3). The bar graphs in Fig. 5C show the top 7 miRNAs that were highly downregulated in culture (as compared to either P11 or adult MG, cutoff 20,000 counts, >10% decline). For example, miR-204, miR-9, and miR-181a, the most highly expressed miRNAs in MG, showed the greatest decline *in vitro*, together with some members of the let-7 family. miR-125-5p and miR-720 are miRNAs which further increased with maturation *in vivo* and also increased their levels *in vitro,* although these levels were lower than those of freshly isolated adult MG (Fig. 5C’). We were specifically interested in the let-7 family of miRNAs, due to the recent demonstration that these are regulated in MG during regeneration in the zebrafish. Several members of the let-7 family, let-7c, let-7b, let-7d, and let-7g were among the most highly expressed miRNAs in P11 or adult MG. Most of them (except let-7e and let-7i) were substantially reduced in the cultured MG when compared with the P11 or adult MG *in vivo* (see heat map), and declined in the cultured 14 DIV MG (Fig. 5D). In zebrafish, let-7 represses the reprogramming of the MG that leads to regeneration, and an increase in the let-7 inhibitor, Lin28, is required for regeneration in zebrafish^4^. The reduction in let-7 levels that occurs in dissociated culture of MG is consistent with the recent observation that mouse MG can be reprogrammed to neurogenic progenitors and neurons *in vitro* more effectively than *in vivo*^6^,^9^.

However, while most miRNAs declined in cultured MG, some showed large increases, such as miR-22 and miR-23a (Fig. 5E, cutoff 5,000 counts, increase >10%). Moreover, there were two miRNAs (miR-21 and miR-145) and three miRNAs (miR-199a-3p/5p and miR-143), which were not or barely expressed *in vivo* (<500 counts), but increased dramatically *in vitro* (over 6,900 and over 1,500 counts respectively, Fig. 5F).

Figure 5G summarizes the changes in the miRNA profile of MG *in vitro* (8 DIV + 14DIV) compared with freshly isolated MG (P11 + adult), as a function of the category of expression. *In vitro*, 72% of all mGliomiRs and 79% of all shared miRs declined (range of decline is given in brackets). Only 28% of all mGliomiRs (i.e., miR-23) and 21% of all shared miRs increased. Interestingly, a few miRNAs barely expressed in MG *in vivo* (<40 counts), increased substantially in cultured MG (>1,500 counts).

## Discussion

Since MG represent only 2–3% of the retina, we optimized methods for purifying the cells with FACS to identify the miRNAs expressed in MG. Using the Rlbp-CreER: tdTomato mice, which has been shown to selectively label MG and no other retinal cell^30^,^31^, we are able to prepare populations of MG that are over 90% pure, similar to levels observed with MACS sorting^16^, and an improvement over previous FACS purified MG using other mouse lines^32^. To assess miRNA expression in the FACS purified cells, we pooled cells from at least 40 retinas. The RNA from both the sorted tdTomato^+^ cells (MG) and the tdTomato^–^ cells (neurons, 90% rods) was then subjected to NanoString nCounter^®^ analysis. Although there are now many platforms for characterizing miRNA expression, NanoString quantitation is somewhat better than microarrays in accuracy and reproducibility (R^2^ = 0.99 and R^2^ = 0.91 respectively), and similar in sensitivity to RT-qPCR^14^, for review see^33^. In addition, the NanoString assay can accurately discriminate miRNAs with similar sequences, such as members of the let-7 family^15^ and does not require amplification.

The results we obtained for purified MG and neurons using NanoStrings were consistent with those obtained for total retina using microarray-based quantitation^10^,^12^; of the 78–408 miRNAs assayed, between 60% and 81% (respectively) of the highly expressed miRNAs in our samples, were also expressed at high levels in these reports. Seven out of 10 miRNAs that were not expressed in the total retinal samples^12^, were also not expressed in our study of purified MG. However, since we separated the MG from the rest of the retinal cells, we can now assign specific miRNAs either to MG or the retinal neurons. For example, miR-125b has been previously reported to be expressed in the retina, but was not known to be specifically expressed in the MG^10^,^12^,^34^. Thus, our results compare well with other techniques for evaluating levels of miRNAs in the retina. The ability to use NanoStrings to evaluate miRNAs in relatively small amounts of RNA thus allows the capability to use FACS-purified populations, and potentially to profile the various subtypes of ocular neurons.

One of the most highly expressed miRNAs in MG, but not in the neuronal cells, is miR-204. Previous studies have shown that this miRNA is expressed in the lens, the ciliary epithelium, and the retina^34^,^35^,^36^,^37^, in cells of the INL^37^,^38^, though it had not previously been localized to the MG. Although miR-204 is expressed in MG, RPE cells and ciliary epithelial cells, it is unlikely that the MG in our study was contaminated by RPE or ciliary epithelial cells, since (1) the FACS-sorted tdTomato^+^ cells in our samples were Sox2^+^ and this is not expressed in either the RPE or ciliary epithelium and (2) miRNAs miR-211, miR-222, and miR-221, are highly expressed in the RPE^39^, but were not highly expressed in the MG-fraction in our study. Thus, miR-204 appears to be expressed in non-neuronal cells in the eye. It will be interesting to determine whether this miRNA has similar targets/functions in the diverse types of non-neuronal retinal cells.

Our data also indicate that some miRNAs might serve general functions for glia. Two of the three most highly expressed miRNAs in the MG are also expressed highly in other types of glia. Next to miR-204, the next most highly expressed MG miRNAs (with less than 20% expression in neurons) were miR-125b, and miR-9. These two miRNAs are also highly expressed in P7 FAC-sorted astrocytes of the forebrain, along with several others of the most highly expressed mGliomiRs (miR-99a, miR-204, miR-135a) and shared miRs (miR-720, let-7b, miR-29a, and miR-30d)^40^. A role for miR-125b and miR-9 has been described for retinal development, particularly in the transition from early (non-gliogenic) to late (gliogenic) progenitors^41^, as well as its role during neurogenesis^42^,^43^. This appears to be true for other regions of the CNS as well, where miR-125b is important for astrocyte development^40^,^44^,^45^,^46^, oligodendrocyte progenitors^47^, and even Schwann cell development in the PNS^48^. Moreover, miR-9 is also required for Bergmann glia development in the cerebellum^49^. Thus, miR-125 and miR-9 appear to have general roles in neural progenitors to convey competency to generate glia.

Do these miRNAs have a role in mature glia? There is much less known about the roles of miRNAs in mature glia. In glioma-derived stem cells, low miR-125 levels lead to increased proliferation due to a change in Lin-28, a target of miR-125b^50^. For the other mGliomiRs, miR-135a^40^,^51^, miR-23a^45^, and miR-99a^40^ have been reported in astrocytes as well as for Schwann cells in the sciatic nerve along with miR-100^48^, indicating that these miRNAs could be common among glia. However, it appears, that every glia type also has a specific highly expressed set of miRNAs, which may contribute to their unique patterns of gene expression.

As noted above, many studies of glia are carried out *in vitro,* but it is not known whether cultured glia are similar to freshly isolated cells with regard to their miRNA profile. Although the cluster analysis demonstrates that the pattern of expression of miRNAs are comparable between freshly isolated MG and cells cultured for one or two weeks, nearly all of the miRNAs that are highly expressed in adult MG *in vivo* were down-regulated *in vitro* within 2 weeks. miRNA expression levels are reduced by as much as 90%, and this occurs as early as 8 DIV, regardless of whether housekeeping genes or 5S ribosomal RNA^52^ are used for normalization. Several previous reports have documented many changes in the profiles of mRNA expression and proteome in cultured MG, compared with the values present in freshly isolated MG^16^,^29^,^53^. Therefore, some of these differences may be due to the changes in miRNAs that occur as the cells adapt to *in vitro* conditions. The widespread reduction in miRNAs that we observe in the cultured MG, as compared with the freshly isolated cells, may relate to the observation that decreased cell contact in a monolayer of cells leads to a reduction in miRNA biogenesis *in vitro*^52^.

Although the majority of miRNAs are expressed at lower levels in the cultured MG, when compared with those *in vivo*, some miRNAs were expressed at higher levels in the cultured cells. One of the miRNAs we found upregulated by over 90% is miR-21, which has been reported to be increased in reactive astrocytes after lesion^54^,^55^ or in glioma^56^. Interestingly, an over-expression study showed miR-21 is implicated in glial hypertrophy^55^. It is possible that the increase in MG size observed *in vitro* is also a result of the increases in miR-21, but future experiments will be needed to test this hypothesis. Another cluster that is highly up-regulated in the cultured MG is miR-143/145. miR-143/145 along with miR-199a (another miRNA that is more highly expressed in cultured MG but absent in MG *in vivo*) have been recently reported to be highly expressed in cultured MG from P8 mice^25^. Moreover, spinal cord injury also induces an increase in miR-145 in astrocytes^57^. Along with these studies of spinal cord injury, miR-145, together with miR-199a, has been reported to play a role in glial tumors, inhibiting migration^58^,^59^, proliferation^60^, and astrogliosis^57^. Interestingly, miR-199a is also reported to block MeCP2 function in the DGCR/Drosha complex^61^. Since MeCP2 is also part of the REST/CoREST complex that regulates the non-neuronal cell state^62^, this could explain the observation that cultured MG often downregulate the expression of mature MG markers such as Cralbp, GS or GLAST.

In summary, our study has for the first time produced a miRNA profile for mature and young MG *in vivo* and MG *in vitro*. We have defined a set of miRNAs that are specific to the MG (with low to no expression in neurons), as well as identified those miRNAs that are shared between retinal neurons and MG. Many of the miRNAs that are highly expressed in MG are also expressed at high levels in astrocytes, and since astrocytes and MG share many of the same genes^32^, it is possible that miRNA targets are also shared between these two types of glia. Deletion of Dicer1 in astrocytes leads to an increase in genes associated with developing cells and a reduction of mature astrocyte markers, like GLAST or Aquaporin4^63^, and it will be interesting to determine whether loss of miRNAs in MG will lead to a similar phenotype.

## Methods

### Animals

All mice were housed at the University of Washington and all experiments were carried out in accordance with University of Washington Institutional Animal Care and Use Committee approved protocols (UW-IACUC). *Rlbp1-CreERT2* mice were derived from plasmid described by Vázquez-Chona *et al*.^17^ and were crossed to *R26-stop-flox-CAG-tdTomato* mice (Jackson Laboratories, also known as Ai14) and will be henceforth referred to as *Rlbp-CreER: Stop*^*f/f*^*-tdTomato* or wild type (wt). For the photoreceptor-Müller glia dual reporter mouse, *Rlbp-CreER: Stop*^*f/f*^*-tdTomato* mice were crossed to *Nrl-GFP* mice (gift from Dr. Swaroop^19^). Genotyping was done using the primers listed in Supplement Table 4. Tamoxifen (Sigma) was administered intraperitoneally at 75 mg/kg in corn oil at P9 + 10 for the P11 analysis and for two consecutive days at ages P > 21 for the adult assay.

### Fluorescence activated cell sorting (FACS)

We dissociated the retinas of 20 mice and removed the ciliary epithelium (CE, Supplement Fig. 2H, line), RPE and the optic nerve and washed the tissue several times. All retinas were confirmed for successful recombination under the fluorescence microscope. For one sort, 6–10 retinas were pooled and dissociated in DNase/Papain (75 μl/750 μl respectively, Worthington) for 20 min at 37 °C on the shaker, triturated, mixed with Ovomucoid (750 μl) to stop digestive reactions, centrifuged for 10 min at 300 × g and resuspended in 600 μl DNase/Ovomucoid/Neurobasal solution (1: 1: 10 respectively, Gibco) per retina. Before sorting cells were filtered through a 35 μm filter to avoid cell clumps. Cells were sorted using an 80 micron nozzle and collected into two separate chilled tubes, one for the tdTomato^+^ MG and one for the tdTomato^−^ neurons. Debris was excluded from the sort and only all events in gate P1 were sorted (Supplement Fig. 1A). Cells with the brightest fluorescence were found in gate P3 (“positives” (+), MG fraction), cells with no fluorescence in gate P2 (“negatives” (−), neuronal fraction, Supplement Fig. 1A’), everything in between was excluded. Samples were collected in bovine serum albumin (BSA) coated tubes containing Neurobasal medium. Cell sorts were performed using of BD Aria III cell sorter (BD Bioscience). After collection, the tdTomato^+^ MG fraction and the tdTomato^−^ fraction were post-sorted to validate purity. In addition, one drop of each condition was plated on coverslips and evaluated with regard to purity. All other cells were spun for 10 min at 300 × g at 4 °C, the pellet was homogenized in Qiazol and stored at −80 °C (Qiagen).

### Müller glia primary culture

Müller glia were dissociated (see above) from a total of 20 postnatal day (P) 11/12 mice (3 independent cultures of 5, 8 and 7 mice) and grown in Neurobasal medium supplemented with N-2, tetracycline-free 10% fetal bovine serum (FBS, Clontech), and epidermal growth factor (EGF, R&D Systems, 100 ng/mL) as described previously^29^. After 6 days *in vitro* (DIV), cells were passaged and two day later either used for the 8 DIV sample or kept for another week in normal medium.

### Fixation, Sectioning, and Immunofluorescent Labeling

Eyes were fixed in 4% paraformaldehyde (PFA) for 30–60 min, treated with 30% sucrose in phosphate buffered saline (PBS) overnight, embedded in OCT embedding medium and cross sectioned in 12 μm thick sections. MG co-cultures were fixed with 2% PFA. For immunofluorescent staining, cells were incubated in blocking solution (5% milk block: 2.5 g nonfat milk powder in 50 mL PBS; with 0.5% Triton-X100) for 1 h at RT. Primary antibodies (Supplement Table 5) were incubated in 5% milk block overnight, secondary antibodies (Invitrogen/Molecular Probes, and Jackson ImmunoResearch, 1:500–1,000) for 1 h at RT and counterstained with 4,6-diamidino-2-phenylindole (DAPI, Sigma, 1:1,000). EdU labeling was carried out using Click-iT EdU Kit (Invitrogen).

### RNA purification and Reverse Transcriptase Quantitative PCR (RT-qPCR)

The sorts of 40 retinas and of 2 independent primary cultures were pooled for the RNA-purification. RNA was extracted and purified with a miRNeasy Micro Kit in accordance with manufacturer’s instructions (Qiagen). For miRNA RT-qPCR, cDNA stem-loop RT primers were used to produce cDNA for specific miRNAs^64^,^65^. Primers are shown in Supplement Table 6. RT-qPCR was performed using SsoFast EvaGreen Supermix (Bio-Rad) on a Bio-Rad Thermocycler for the MG-enriched and the neuronal FAC-sorted fractions. Reactions were run in triplicates or duplicates for RT or –RT samples. Values were normalized to *5sRNA* and or β*-actin*. Delta delta C_t_ between adult and young MG were calculated and were expressed together with the standard deviation (S.D.).

### NanoString Technologies^®^ to profile miRNAs

NanoString nCounter^®^ was used for miRNA expression analysis. DNA sequences called miRtags were ligated to the mature miRNAs through complementary oligonucleotides with sequence-specific binding (bridges). All excess tags and bridges were removed, resulting in sequence-specific tagging of mature miRNAs. The miRtagged mature miRNA was then hybridized to a probe pair (reporter probe and capture probe) in the standard nCounter gene expression array workflow. 200 ng of total RNA per sample (33 ng/μl, n = 20 mice for adult MG, n = 16 mice for P11 MG, n = 13 mice for cell culture, pooled samples) was submitted for NanoString analysis, performed at the Fred Hutchinson Cancer Center in Seattle, WA, USA. NanoString data was analyzed using nSolver 2.6 software. The data represents counts of molecules normalized against 4 housekeeping genes (β*-actin*, *GAPDH*, *Rpl19*, and *B2m*), 8 negative controls, and 6 positive controls that were run simultaneously with the samples.

### Microscopy, Cell Counts, and Statistical Analysis

Live imaging was performed using Zeiss Observer D1 with Axio-Cam. Fixed cells were analyzed by Olympus FV1000 confocal microscope. For cell cultures/plated cells, six random fields per coverslip, at 200x magnification were counted for every condition. For retinal cross sections, two areas/section with 625 μm × 625 μm dimension at 200x magnification, 4 optical sections of 2 μm thickness, for 5 non-consecutive sections per mouse, of 6 adult or 4 young mice were counted. Values are expressed as mean ± standard deviation (S.D.). Statistical analyses were performed by Student’s t-test for independent samples combined with Levene’s test for equality of variances. Holm-Bonferroni method was used to correct for multiple comparisons.

## Additional Information

**How to cite this article**: Wohl, S. G. and Reh, T. A. The microRNA expression profile of mouse Müller glia *in vivo* and *in vitro*. *Sci. Rep.* **6**, 35423; doi: 10.1038/srep35423 (2016).

## Supplementary Material

Supplementary Information
