# Supplementary material for: The microRNA expression profile of mouse Müller glia in vivo and in vitro
Source: Sci Rep. 2016 Oct 14;6:35423. doi: 10.1038/srep35423 (PMC5064377; doi:10.1038/srep35423)
Supplement: Supplementary Information [file srep35423-s1.pdf]

Supplemental Files for:

The microRNA expression profile of mouse Müller glia *in vivo* and *in vitro.*

Stefanie G. Wohl and Thomas A. Reh

Department of Biological Structure, University of Washington, School of Medicine, Seattle, WA, USA

Correspondence: tomreh@uw.edu

Supplemental Figures 1-3

Supplemental Tables 1-6

Supplement Figure 1. Purity of FAC-sorted cells from adult Rlbp-CreER: Stop$^{f/f}$-tdTomato mice. A-A''': Gating for FACS and proportions of sorted cells for adult retinas. A: all cells in gate P1 (blue dots) have been sorted based on their size (forward scattered, FSC) and fluorescence/granularity (side scattered, SSC). Very small cells and debris (black dots) have been excluded. A': tdTomato^{+} cells were found in gate P3 (brightest red fluorescence), while the vast majority of tdTomato^{-} cells were found in gate P2 (no red fluorescence). The percentage of the P2 and P3 events of either P1 or total events or are shown in A''. A''' shows the fraction of the sorted tdTomato^{+} cells in the SSC-A and FSC-A scheme. **B, D:** Immunofluorescent labeling with antibodies against tdTomato, Sox9 (B), Sox2 (D) and DAPI nuclear staining of the MG-enriched tdTomato^{+} FAC-sorted fractions, plated on a feeder layer of young glia, after 8 days *in vitro* (DIV). **C, E:** Percentage of tdTomato^{+}Sox9^{+} (C) or tdTomato^{+}Sox2^{+} (E) cells found in the P3-gated (tdTomato^{+} MG) and P2-gated (tdTomato^{-} neuronal) FAC-sorted fractions of 2 mice. **F-H:** Immunofluorescent labeling with antibodies against tdTomato and Sox2 and DAPI nuclear staining of a periphery of a retinal cross section (adult). **I:** Retinal cross section of a Rlbp-CreER-tdTomato: Nrl-GFP mouse. **J-K:** Live images of plated tdTomato^{+} Nrl-GFP^{-} cells (MG, C, C') and tdTomato^{-} Nrl-GFP^{+} cells (neurons/rods, D, D') after FACS. ONL: outer nuclear layer, OPL: outer plexiform layer, INL: inner nuclear layer, IPL: inner plexiform layer, GCL: ganglion cell layers, RPE: retinal pigment epithelium, CE: ciliary epithelium.

Supplement Figure 2. Purity of FAC-sorted cells from P11 Rlbp-CreER: Stop$^{f/f}$-tdTomato mice. A-A'': Gating for FACS and proportions of sorted cells from P11 retinas: all cells in gate P1 (blue dots) have been sorted based on their size (forward scattered, FSC) and fluorescence/granularity (side scattered, SSC). Very small cells and debris (black dots) have been excluded. tdTomato^{+} cells were found in gate P3 (brightest fluorescence), while the vast majority of tdTomato^{-} cells were found in gate P2 (no fluorescence, A'). The

percentage of the P2- and P3-gated events of either P1 or total events or are shown in A''. A''' shows the fraction of the sorted tdTomato^{+} cells in the SSC and FSC-A scheme. **B-E:** Live images of cultured P11 FAC-sorted tdTomato^{+} (MG, B, B, D, D') and tdTomato^{-} cells (neurons, C, C', E, E') after the sort (B, C) and after 5 DIV (D, E). **F:** Number of tdTomato^{+} cells per field found in the tdTomato^{+} (MG) and tdTomato^{-} (neurons) cultured fractions 5 days after FACS. **G, H:** Immunofluorescent labeling of tdTomato^{+} cells with Id1 and DAPI (G) and Sox2 and DAPI (H) to confirm glial identity (from 2 independence sorts, total n = 6 mice). **I:** Percentage of tdTomato^{+}Id1^{+} cells of total Id1^{+} or DAPI^{+} cells. **J:** Percentage of tdTomato^{+}Sox2^{+} cells of total Sox2^{+} or DAPI^{+} cells. Significant differences are indicated, **: $p > 0.01$, Student's t-test for independent samples and Levene's test for equality of variances.

Supplement Figure 3: Most MG miRNAs decline in the early culture period. A. Scatter plot of Log2 counts of the miRNAs expressed in P11 MG and 8 DIV MG. The red dots represent miRNAs expressed higher in P11 MG *in vivo* than in 8 DIV MG, the black dots miRNAs higher expressed in 8 DIV MG than P11 MG *in vivo*. **B, C:** Bar graphs of 4 mGliomiRs which expression levels decreased after 8 DIV as compared to P11 MG *in vivo* (B) and of 3 mGliomiRs which expression levels increased after 8 DIV as compared to P11 MG *in vivo* (C). **D, E:** Bar graphs of 10 shared miRs in which expression levels decreased after 8 DIV as compared to P11 MG *in vivo* (D) and of 3 shared miRs in which expression levels increased after 8 DIV as compared to P11 MG *in vivo* (E). Bar graphs cutoff 20,000 counts.

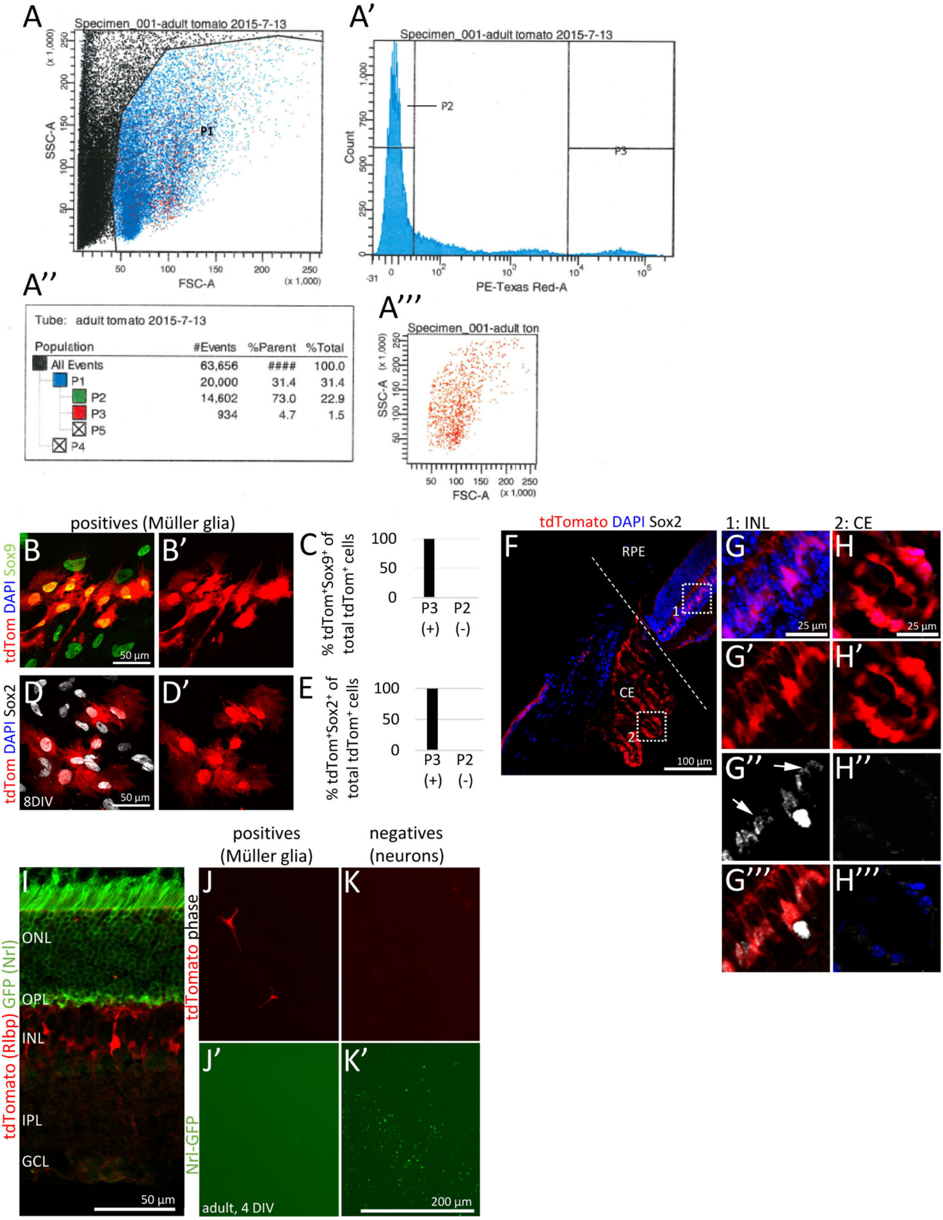

A
Specimen_001-adult tomato 2015-7-13
SSC-A
FSC-A
P1
A'
Specimen_001-adult tomato 2015-7-13
Count
PE-Texas Red-A
P2
P3
A''
Tube: adult tomato 2015-7-13
Population #Events %Parent %Total
All Events 63,656 #### 100.0
P1 20,000 31.4 31.4
P2 14,602 73.0 22.9
P3 934 4.7 1.5
P5
P4
A'''
Specimen_001-adult ton
SSC-A
FSC-A
positives (Müller glia)
B B' C D D' E
tdTom DAPI Sox9
tdTom DAPI Sox2
8DIV
50 µm
% tdTom+Sox9+ of total tdTom+ cells
% tdTom+Sox2+ of total tdTom+ cells
P3 (+) P2 (-)
tdTomato DAPI Sox2
F
RPE
CE
100 µm
1: INL
2: CE
G G' G'' G''' H H' H'' H'''
25 µm
positives (Müller glia)
negatives (neurons)
I
ONL
OPL
INL
IPL
GCL
tdTomato (Rlbp) GFP (Nrl)
50 µm
J J' K K'
tdTomato phase
Nrl-GFP
adult, 4 DIV
200 µm

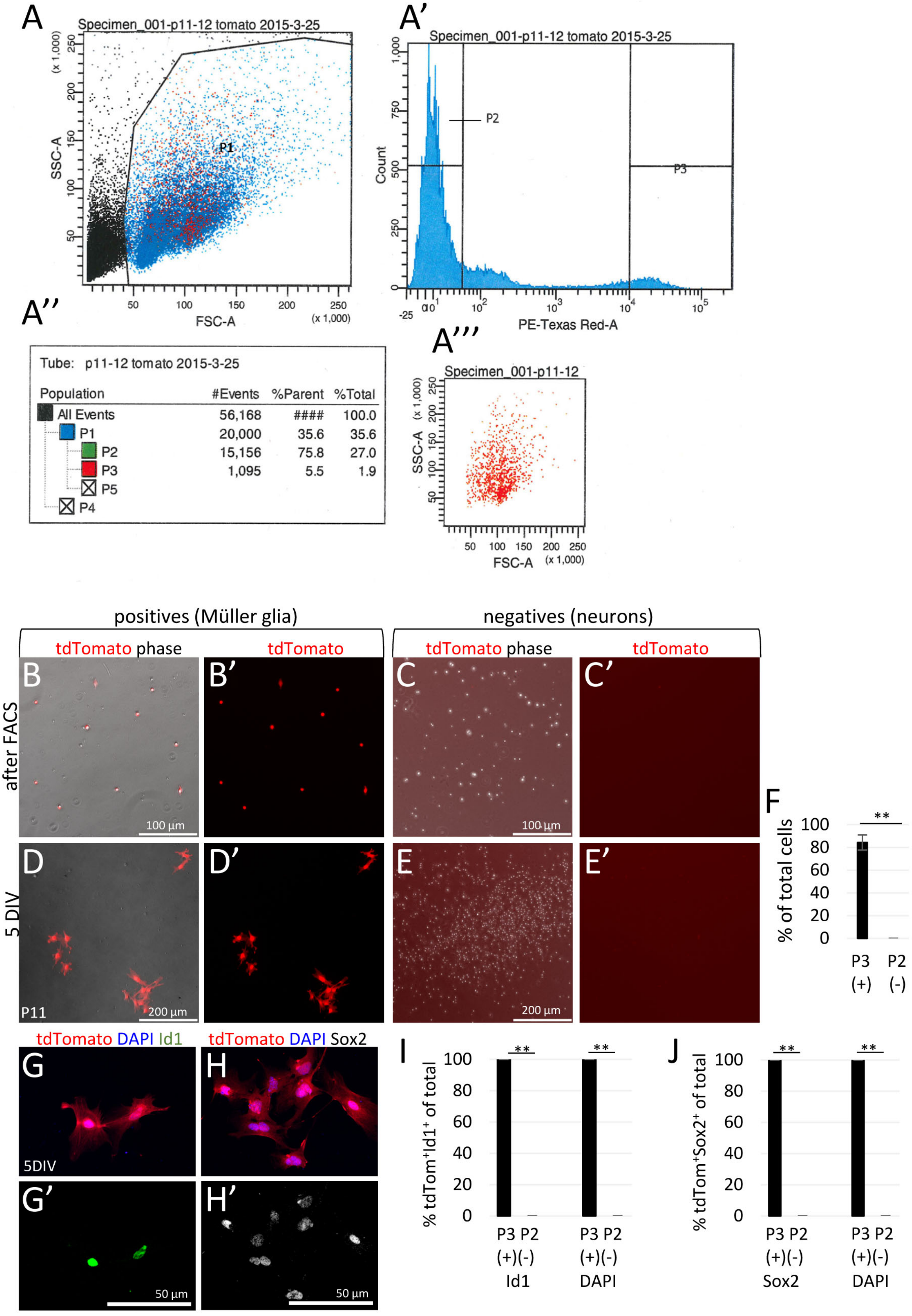

A
Specimen_001-p11-12 tomato 2015-3-25
SSC-A
FSC-A
(x 1,000)
P1
A’
Specimen_001-p11-12 tomato 2015-3-25
Count
PE-Texas Red-A
P2
P3
A’’
Tube: p11-12 tomato 2015-3-25
Population #Events %Parent %Total
All Events 56,168 #### 100.0
P1 20,000 35.6 35.6
P2 15,156 75.8 27.0
P3 1,095 5.5 1.9
P5
P4
A’’’
Specimen_001-p11-12
SSC-A
FSC-A
(x 1,000)
positives (Müller glia)
negatives (neurons)
tdTomato phase
tdTomato
after FACS
5 DIV
B
B’
C
C’
D
D’
E
E’
P11
100 µm
200 µm
F
% of total cells
P3 (+)
P2 (-)
**
tdTomato DAPI Id1
tdTomato DAPI Sox2
G
G’
H
H’
5DIV
50 µm
I
% tdTom+Id1+ of total
P3 P2 (+)(-) Id1
P3 P2 (+)(-) DAPI
J
% tdTom+Sox2+ of total
P3 P2 (+)(-) Sox2
P3 P2 (+)(-) DAPI

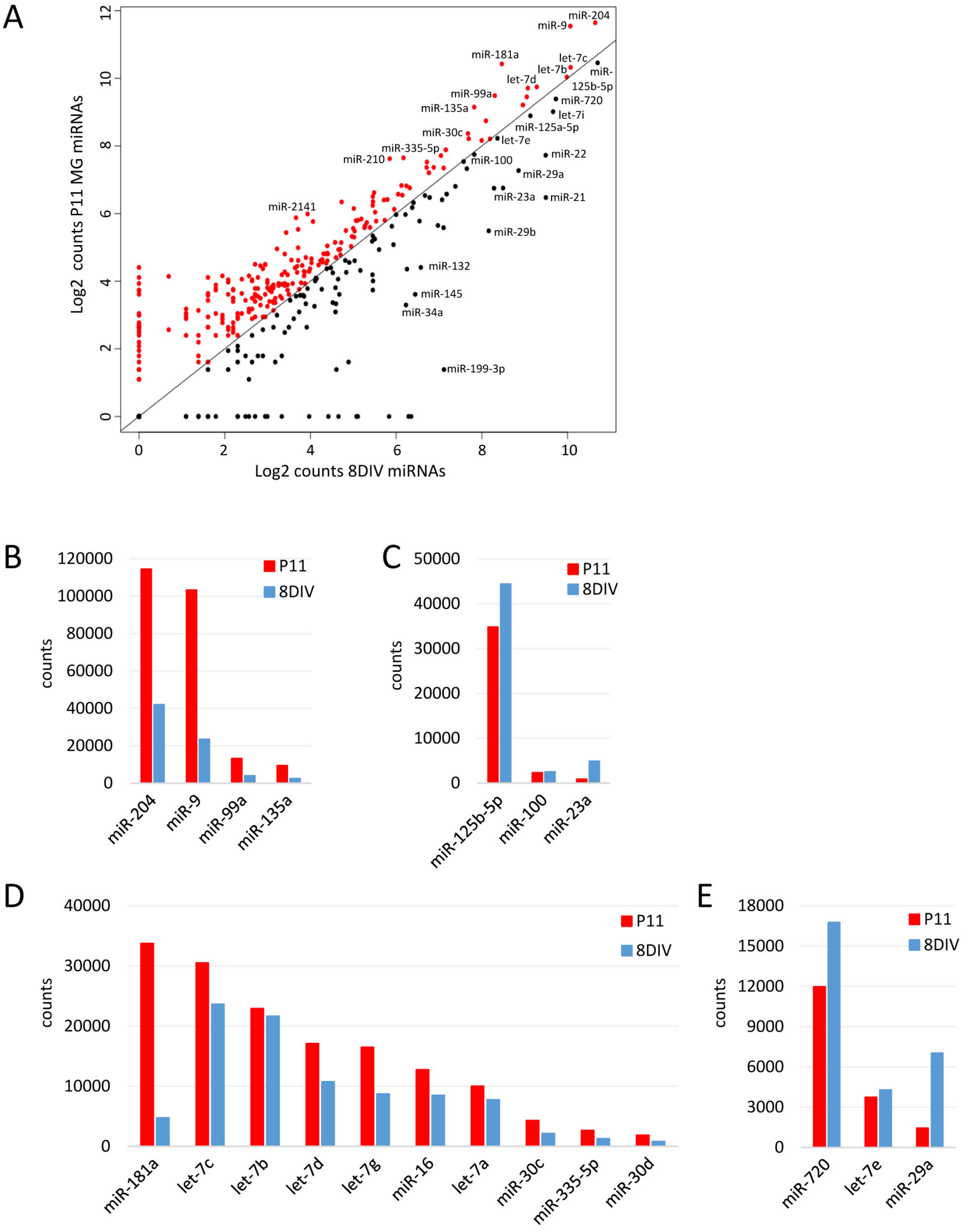
A
Log2 counts P11 MG miRNAs
Log2 counts 8DIV miRNAs
miR-204
miR-9
miR-181a
let-7c
let-7b
miR-125b-5p
let-7d
miR-99a
miR-720
miR-135a
let-7i
miR-125a-5p
miR-30c
let-7e
miR-335-5p
miR-22
miR-210
miR-100
miR-29a
miR-23a
miR-21
miR-2141
miR-29b
miR-132
miR-145
miR-34a
miR-199-3p
B
counts
P11
8DIV
miR-204
miR-9
miR-99a
miR-135a
C
miR-125b-5p
miR-100
miR-23a
D
miR-181a
let-7c
let-7b
let-7d
let-7g
miR-16
let-7a
miR-30c
miR-335-5p
miR-30d
E
miR-720
let-7e
miR-29a

Supplemental Table 1: FACS data - input and purity.

sample	# of sorts	# of retinas	# of cells in suspension “positives“	postsort purity “positives“	% of total P1 events	% of total events	# of cells in suspension “negatives“	postsort purity “negatives“
adult	6	40	4444839	96%	5%	1.8%	56.5×10^6	0%
P11/12	5	32	3378620	95%	4%	2.1%	29.7×10^6	0%

Supplemental Table 2: Expression levels of the highly expressed 23 miRNAs (cutoff 2000 counts) in adult Müller glia compared to adult neurons and young Müller glia (mGliomiRs are bold).

miRNA	Müller glia (MG) adult (counts)	Neurons adult (counts)	adult MG vs. neurons (fold)	adult neurons vs. MG (fold)	Müller glia P11/12 (counts)	% increase P11 to adult
**mmu-miR-204**	**213460**	**11740**	**18**	**0**	**62946**	**71**
**mmu-miR-125b-5p**	**81882**	**9291**	**9**	**0**	**19159**	**77**
**mmu-miR-9**	**56346**	**8338**	**7**	**0**	**56806**	**-1**
mmu-miR-181a	36482	69753	1	2	18556	49
mmu-let-7c	19558	11497	2	1	16782	14
mmu-miR-720	17859	15686	1	1	6585	63
**mmu-miR-99a**	**14804**	**2394**	**6**	**0**	**7259**	**51**
mmu-let-7b	13480	12275	1	1	12600	7
mmu-let-7g	11687	41497	0	4	9065	22
mmu-miR-30c	10425	22609	0	2	2367	77
mmu-let-7d	9932	26367	0	3	9405	5
**mmu-miR-135a**	**9588**	**1148**	**8**	**0**	**5173**	**46**
mmu-miR-29a	6511	4391	1	1	792	88
mmu-let-7a	6073	11665	1	2	5513	9
mmu-miR-16	5424	27745	0	5	7007	-29
mmu-miR-125a-5p	4213	7019	1	2	4001	5
**mmu-miR-100**	**4203**	**2**	**2102**	**0**	**1272**	**70**
mmu-miR-1944	3753	32472	0	9	3448	8
mmu-miR-22	3672	7435	0	2	1244	66
mmu-miR-30d	3493	3976	1	1	1019	71
mmu-let-7e	2831	5004	1	2	2057	27
mmu-miR-335-5p	2487	2568	1	1	1464	41
**mmu-miR-23a**	**2485**	**374**	**7**	**0**	**472**	**81**

Supplemental Table 3: Expression levels of the highly expressed 46 neuronal miRNAs (cutoff 2000 counts) in adult neurons compared to adult Müller glia and young neurons (17 miRNAs with expression levels in MG <20% are bold).

miRNA	Neurons adult (counts)	MG glia adult (counts)	MG fold vs neurons (adult)	Neurons fold vs MG (adult)	% expression in adult MG	Neurons young (counts)	% increase from P11 to adult in neurons
**mmu-miR-124**	**308141**	**1011**	**0**	**305**	**0.3**	**39060**	**87**
**mmu-miR-183**	**101370**	**271**	**0**	**374**	**0.3**	**12473**	**88**
**mmu-miR-96**	**87840**	**212**	**0**	**414**	**0.2**	**16891**	**81**
mmu-miR-181a	69753	36482	1	2	52	40225	42
mmu-let-7g	41497	11687	0	4	28	15320	63
**mmu-miR-1944**	**32472**	**3753**	**0**	**9**	**12**	**2813**	**91**
mmu-miR-16	27745	5424	0	5	20	7497	73
mmu-let-7d	26367	9932	0	3	38	9005	66
mmu-miR-30c	22609	10425	0	2	46	4928	78
**mmu-miR-29c**	**19530**	**1600**	**0**	**12**	**8**	**949**	**95**
mmu-miR-720	15686	17859	1	1	114	4077	74
mmu-let-7b	12275	13480	1	1	110	5918	52
mmu-miR-204	11740	213460	18	0	1818	7275	38
mmu-let-7a	11665	6073	1	2	52	5499	53
mmu-let-7c	11497	19558	2	1	170	7258	37
**mmu-miR-211**	**11181**	**250**	**0**	**45**	**2**	**2732**	**76**
mmu-miR-125b-5p	9291	81882	9	0	881	3047	67
**mmu-miR-129-3p**	**8723**	**236**	**0**	**37**	**3**	**357**	**96**
mmu-miR-9	8338	56346	7	0	676	15876	-90
mmu-miR-25	8203	1761	0	5	21	5420	34
mmu-miR-22	7435	3672	0	2	49	1343	82
mmu-miR-342-3p	7103	1827	0	4	26	1606	77
mmu-miR-125a-5p	7019	4213	1	2	60	5689	19

mmu-let-7f	5850	1718	0	3	29	2539	57
**mmu-miR-151-5p**	**5273**	**942**	**0**	**6**	**18**	**748**	**86**
**mmu-miR-1186**	**5130**	**175**	**0**	**29**	**3**	**638**	**88**
mmu-let-7e	5004	2831	1	2	57	1865	63
**mmu-miR-182**	**4959**	**1**	**0**	**4959**	**0.02**	**1039**	**79**
**mmu-miR-451**	**4684**	**1**	**0**	**4684**	**0.02**	**340**	**93**
mmu-miR-29a	4391	6511	1	1	148	127	97
mmu-miR-30d	3976	3493	1	1	88	835	79
**mmu-miR-210**	**3767**	**566**	**0**	**7**	**15**	**858**	**77**
mmu-let-7i	3516	1820	1	2	52	3958	-13
**mmu-miR-500**	**3073**	**1**	**0**	**3073**	**0.03**	**534**	**83**
mmu-miR-15b	3001	697	0	4	23	2815	6
**mmu-miR-1224**	**2828**	**129**	**0**	**22**	**5**	**338**	**88**
**mmu-miR-301a**	**2750**	**534**	**0**	**5**	**19**	**895**	**67**
mmu-miR-1937a+b	2681	817	0	3	30	361	87
**mmu-miR-15a**	**2571**	**376**	**0**	**7**	**15**	**2011**	**22**
mmu-miR-335-5p	2568	2487	1	1	97	773	70
mmu-miR-191	2538	671	0	4	26	337	87
mmu-miR-99a	2394	14804	6	0	618	1691	29
mmu-miR-26b	2320	466	0	5	20	346	85
mmu-miR-30a	2320	1612	1	1	69	318	86
**mmu-miR-132**	**2018**	**88**	**0**	**23**	**4**	**257**	**87**
mmu-miR-148a	2015	1598	1	1	79	685	66

Supplemental Table 4: genotyping primers

Gene name	Forward sequence (5' to 3')	Reverse sequence (3' to 5')
Rlbp1Cre transgene	CAAGTGTGAGAGACAGCATTG	TCCTTAGCGCCGTAAATCAA
tdTomato wildtype	AAG GGA GCT GCA GTG GAG TA	CCG AAA ATC TGT GGG AAG TC
tdTomato mutant	CTG TTC CTG TAC GGC ATG G	GGC ATT AAA GCA GCG TAT CC
Nrl-GFP	TGG AAA CCA AGC TCA CAA CA	GAA CTT CAG GGT CAG CTT GC

Supplemental Table 5: primary antibodies

**antibody**	**concentration**	**Company, Catalog #**
rat anti RFP (tdTomato)	1:500	Antibodies online, ABIN334653
mouse anti glutamine synthetase (GS)	1:200	Millipore, MAB 302
rabbit anti Sox9	1:1000	Millipore, AB5535
goat anti Sox2 (Y-17)	1:100	Santa Cruz, sc-17320
Rabbit anti Id1	1:1000	BioCheck, BCH-1/37-2
chicken anti GFP	1: 500	Abcam, ab13970

Supplemental Table 6: RT-qPCR primers for miRNAs

miRNA	Forward sequence (5' to 3')	Reverse sequence (3' to 5')	Stem loop RT primer (3' to 5')
miR-204	CGC GCT TCC CTT TGT CAT CC	TCG GAG CGC CAA GTG TAC AG	TGA GCA GCG CTC GGA GCG CCA AGT GTA CAG GCG CTG CTC AAG GCA TAG
miR-125b-5p	CCG CTC CCT GAG ACC CTA A	CGA AGG AAC TTG GGA TAT GAC G	ATA GGG GCG CGA AGG AAC TTG GGA TAT GAC GCG CCC CTA TTC ACA AGT
miR-9	GCC GGT CTT TGG TTA TCT AGC	TTT CCT CGA CCG CAC CAC TC	AAA TCG CAG CTT TCC TCG ACC GCA CCA CTC GCT GCG ATT TTC ATA CAG
miR-181b	GCG CCA ACA TTC ATT GCT GTC	GAT CAA GGG GCA GCA CGT AG	GGC TAG CAG CGA TCA AGG GGC AGC ACG TAG GCT GCT AGC CAC CCA CCG
miR-9	GCC GGT CTT TGG TTA TCT AGC	TTT CCT CGA CCG CAC CAC TC	AAA TCG CAG CTT TCC TCG ACC GCA CCA CTC GCT GCG ATT TTC ATA CAG
let-7c	CGG CCT TGA GGT AGT AGG TT	GCT TCG CTC TTA TTT CCT GAT GG	CGC TAC AGG CGC TTC GCT CTT ATT TCC TGA TGG GCG CGA TGT CAA CCA TAC
5s	GCC ATA CCA CCC TGA ACG	TGC AGG GTC CGA GGT ATT CG	ACT GCT GCG CTG CAG GGT CCG AGG TAT TCG GCG CAG CAG TAA AGC CTA
